# 260 fs, 403 W coherently combined fiber laser with precise high-order dispersion management

**DOI:** 10.1007/s12200-024-00107-5

**Published:** 2024-01-22

**Authors:** Shuangxi Peng, Zhihao Wang, Feilong Hu, Zhengyan Li, Qingbin Zhang, Peixiang Lu

**Affiliations:** 1 Wuhan National Laboratory for Optoelectronics and School of Physics, Huazhong University of Science and Technology, Wuhan, 430074 China; 2 School of Optical and Electronic Information, Huazhong University of Science and Technology, Wuhan, 430074 China; 3 Optics Valley Laboratory, Wuhan, 430074 China

**Keywords:** Fiber lasers amplifier, Power scaling, Coherent beam combination, Dispersion compensation

## Abstract

An ultrafast fiber laser system comprising two coherently combined amplifier channels is reported. Within this system, each channel incorporates a rod-type fiber power amplifier, with individual operations reaching approximately 233 W. The active-locking of these coherently combined channels, followed by compression using gratings, yields an output with a pulse energy of 504 μJ and an average power of 403 W. Exceptional stability is maintained, with a 0.3% root mean square (RMS) deviation and a beam quality factor *M*^2^ < 1.2. Notably, precise dispersion management of the front-end seed light effectively compensates for the accumulated high-order dispersion in subsequent amplification stages. This strategic approach results in a significant reduction in the final output pulse duration for the coherently combined laser beam, reducing it from 488 to 260 fs after the gratings compressor, while concurrently enhancing the energy of the primary peak from 65% to 92%.

**Graphical Abstract:**

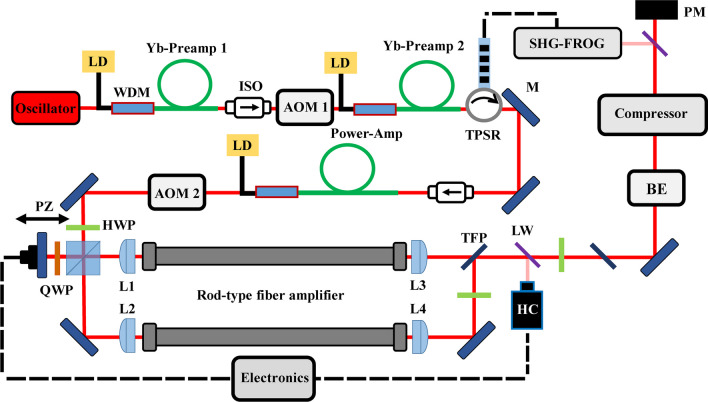

## Introduction

High-power ultrafast lasers are indispensable tools in various fields, spanning fundamental and applied scientific research to industrial processing [1–4]. Thin disk [5], slab [6], and fiber [7] lasers utilize distinctive geometries and effective heat dissipation to achieve remarkable average powers. Fiber lasers, in particular, can be a compelling choice due to inherent advantages such as scalable power, exceptional beam quality, advanced thermal management, and consistent stability. Despite these commendable features, fiber lasers encounter two primary limitations hindering further power scaling: nonlinearity and transverse mode instability (TMI) [8]. To address the first of these challenges, large mode field area optical fibers are commonly employed to mitigate nonlinear effects. However, this approach can lead to the emergence of high-order modes, potentially resulting in reduced beam quality. A rod-type photonic crystal fiber (rod-type PCF) based fiber amplifier is a method that can significantly increase the average power and pulse energy. The substantial mode field area and relatively short length (typical 50–100 cm) of rod-type PCF efficiently suppress nonlinear effects, while also ensuring near-single-mode beam transmission within the fiber. The unique photonic crystal structure of rod-type PCF also effectively suppresses TMI, thus addressing the second issue. Rod-type PCF has been successfully applied in fiber amplifiers that generate average powers in the range of hundred watts [9–11] and pulse energies at the millijoule level [12, 13], enabling various high-power laser applications. NKT Photonics A/S recently reported achieving pulses with an average power of 248 W in a newly designed rod-type PCF with enhanced TMI suppression, thereby extending the power scaling boundary of a single rod-type fiber [14].

Nevertheless, rod-type PCF still grapples with inherent power scaling limitations stemming from the self-focusing effect. For fiber laser operating in the fundamental transverse mode, this effect constrains the attainable peak power to a range of 4–6 MW at 1 μm [15]. In pursuit of further power scaling, a successful strategy to overcome this constraint lies in coherent beam combination (CBC). Under the conditions of mutual coherence and stable phase relationship, multiple laser beams can be superimposed and mutually interfere with each other. This approach allows for an improvement in average power and pulse energy by a factor almost equal to the total number of combined channels. Utilizing two-channel CBC technology, the average power of the fiber laser reaches approximately 100 W, with a corresponding pulse energy of a hundred microjoules. The escalation in combined channels correlates with a proportional rise in combined average power, increasing from 100 W to the kilowatt range [16–20], accompanied by an increase in pulse energy from 100 μJ to 30 mJ [8, 12, 16, 17, 21–24]. These CBC systems exhibit a single-channel power output of approximately 100 W, with the required number of combining channels being a multiple of 100 W. The growth in channel count introduces complexity of the CBC system, resulting in a reduction in beam combining efficiency, beam quality, stability, and other performance factors. Consequently, there is a pressing need to increase the power per channel, while simultaneously reducing the number of combining channels for optimal system performance. To attain simultaneous high peak-power, CBC technology is often integrated with chirped-pulse amplification (CPA). This approach involves stretching the pulses to several nanoseconds, allowing seed light to be almost linearly amplified to average power levels near a hundred watts. The amplified pulses are then compressed using a pair of diffraction gratings. However, due to gain narrowing and dispersion mismatch, the compressed pulses typically fall short of the Fourier transform-limited pulse width, often being compressed to around 300 fs [8, 21]. Although further pulse duration compression is possible through spectral shaping and post-compression techniques [12], these approaches notably increase the system’s complexity and operational challenges. The need for a simpler pulse compression solution persists in the field. Furthermore, addressing the challenge of achieving high average power and pulse energy with fewer beam combining channels is also crucial.

This study introduces a high-power, high-energy fiber laser system utilizing CBC technology with two ytterbium-doped rod-type fiber amplifiers. With the achievement of an average power of over 230 W for a single fiber amplifier, while concurrently maintaining superior beam quality, the CBC system has been meticulously designed with only two channels. This technological innovation culminates in a remarkable average power output of 433 W, coupled with a stability of 0.3% RMS, effectively overcoming the limitations associated with a single fiber amplifier. To address residual dispersion, particularly high-order dispersion, in this study we employ a homemade second harmonic generation frequency-resolved optical gating (SHG-FROG) for dispersion characterization. Furthermore, we precisely compensate for dispersion up to the fourth order using a tunable pulse stretcher (TPSR). By incorporating these advanced techniques, our two-channel CBC fiber laser system achieves impressive performance, delivering an average power of 403 W, pulse energy of 504 μJ, and pulse duration of 260 fs.

## Experiment

**Fig. 1:**
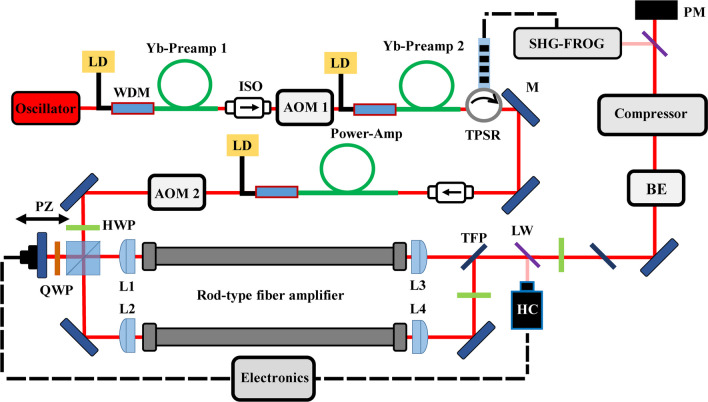
Schematic overview of the experimental setup. *LD* Laser diode, *WDM* Wavelength division multiplexer, *ISO* Isolator, *AOM* Acousto-optic modulator, *TPSR* Tunable pulse stretcher, *M* Reflectivity mirror, *HWP* Half-wave plate, *QWP* Quarter-wave plate, *PZ* Piezo-driven mirror, *L1, L2* Seed light coupling lens, *L3, L4* Pump light coupling and seed light collimating lens, *TFP* Thin film polarizer, *LW* Laser window, *BE* Beam expander, *PM* Power meter

Figure 1 shows the experimental setup, which consists of four parts: fiber laser front-end, ytterbium-doped rod-type fiber amplifier, beam combination, and compressor. The system’s front-end comprises a mode-locked oscillator generating femtosecond pulses at a 40 MHz repetition rate and a 1035 nm center wavelength. Initially, these pulses undergo pre-amplification through Yb-doped single-mode fibers, pumped by a 976 nm fiber-coupled diode laser. An acousto-optic modulator (AOM 1) then reduces the repetition rate from 40 MHz to 800 kHz. To offset losses from AOM 1 and boost seed energy for the high-power amplifiers, we use Yb-doped pre-amplifiers (Yb-Preamp 2).

The tunable pulse stretcher (TPSR) integrates one or two fiber Bragg gratings (FBGs) and all the control electronics to manage the group delay functions of the stretcher. By changing temperature distribution along the TPSR, its dispersion characteristics are modified. Tuning the third-order and even higher-order dispersion parameters minimizes residual uncompensated spectral phase after compression by the gratings compressor. The TPSR in this work allows for adjustments of group delay dispersion (GDD) between − 20 and 20 ps^2^, third-order dispersion (TOD) between − 1 and 1 ps^3^, and fourth-order dispersion (FOD) between − 1 and 1 ps^4^. We utilize the TPSR to extend the seed light duration to approximately 400 ps, a measure crucial for ensuring safety during the subsequent power amplification process. In the final stage, the seed light’s average power is amplified to 40 W. To regulate the output power proportion of the seed light, an acousto-optic modulator (AOM 2) is employed.

In the experiment, the laser front-end’s full power output is harnessed, supplying sufficient seed power to ensure amplification in rod-type fiber amplifiers. After splitting the seed light into two channels by a half-wave plate and a polarizing beam splitter cube (PBS), the transmission light (p-polarized beam) is injected into the amplifier through a mirror. To match the optical path lengths in the two channels and compensate for fluctuations, an additional adjustable delay line is required. Therefore, the s-polarized beam, which is reflected at the PBS, is back reflected at a piezo-mounted mirror. A double-pass through a quarter-wave plate results in the p-polarized beam passing straight through the polarizing beam splitter cube and then being coupled with the amplifier. These main amplifiers, one in the channel, are ytterbium-doped rod-type fibers with a length of 80 cm each. These rod-type fibers have a mode field area of up to 3300 μm^2^, effectively suppressing nonlinear effects during the power amplification process. To ensure safety at high power, both amplifiers are installed with water cooling and operate at 22 °C. Two fiber-coupled pump diodes, each specified to deliver up to 350 W average power at a wavelength of 976 nm, are used to pump the main amplifiers individually. Thereafter, the single output beams are polarization beams combined using the TFP (thin film polarizer) after being collimated. In order to achieve better beam combining efficiency, high-precision spatial overlap of two beams at the TFP is necessary. A deviation from perfectly linearly polarized incident pulses and imperfections of the TFP itself result in power leakage at the other end, therefore causing a reduction of the maximum achievable power of the combined beam. Behind the combining TFP, an antireflection-coated laser window is inserted. A small reflected fraction of the combined beam is routed to a Hänsch–Couillaud (HC) detection system [25]. The HC detection system consists of a quarter-wave plate in front of a polarizing beam splitter with two photodiodes measuring the two output signals. The difference between the two signals is used to calculate the phase difference between the two channels. To provide a feedback signal for the piezo stage, a proportional-integral-derivative controller is used.

For pulse compression in the fiber laser system, a gratings compressor employing highly efficient transmission gratings (groove density of 1740 lines/mm) is used. By adjusting the incident angle of the compressor gratings, the dispersion mismatch between the stretcher unit and compressor is well compensated. Finally, we successfully achieve a compression efficiency of up to 93%. Following compression, a fraction of the combined compressed beam is directed towards SHG-FROG using a laser window, while the remaining power is directed to a power meter for power measurement. The SHG-FROG measures the spectral phase of the combined compressed pulse, facilitating the analysis of residual dispersion information. Ultimately, we intend to utilize this dispersion data, along with TPSR, for precise dispersion compensation, thereby optimizing pulse duration and quality.

## Result and discussion

**Fig. 2:**
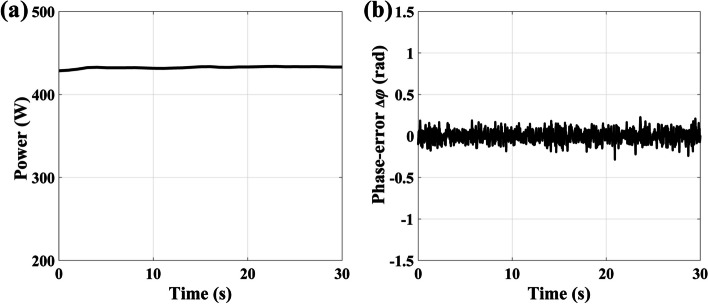
**a** Combined average power is measured over a timescale of 30 s revealing an RMS value of 0.3%. **b** Phase-error Δ*φ* is measured over a timescale of 30 s revealing an RMS value of 67 mrad corresponding to optical length difference fluctuation of *λ*/94

The coupling of seed light is crucial for the entire fiber laser system, as it not only determines the subsequent amplification process, but is crucial to avoid potential damage to the rod-type fiber. The average power of the two channels reaches 233 W when the pump power is 350 W by continuously optimizing the coupling of the seed and pump light. Figure 2 shows the combined beam average power and residual phase error Δ*φ* over a timescale of 30 s during the active phase control. The combined average power root mean square (RMS) is 0.3%, and the residual phase-error Δ*φ* RMS is 67 mrad, which corresponds to an optical path length fluctuation of only *λ*/94. With a beam combining efficiency as high as 93%, we obtain a combined beam average power of 433 W, corresponding to a pulse energy of 541 μJ.

**Fig. 3:**
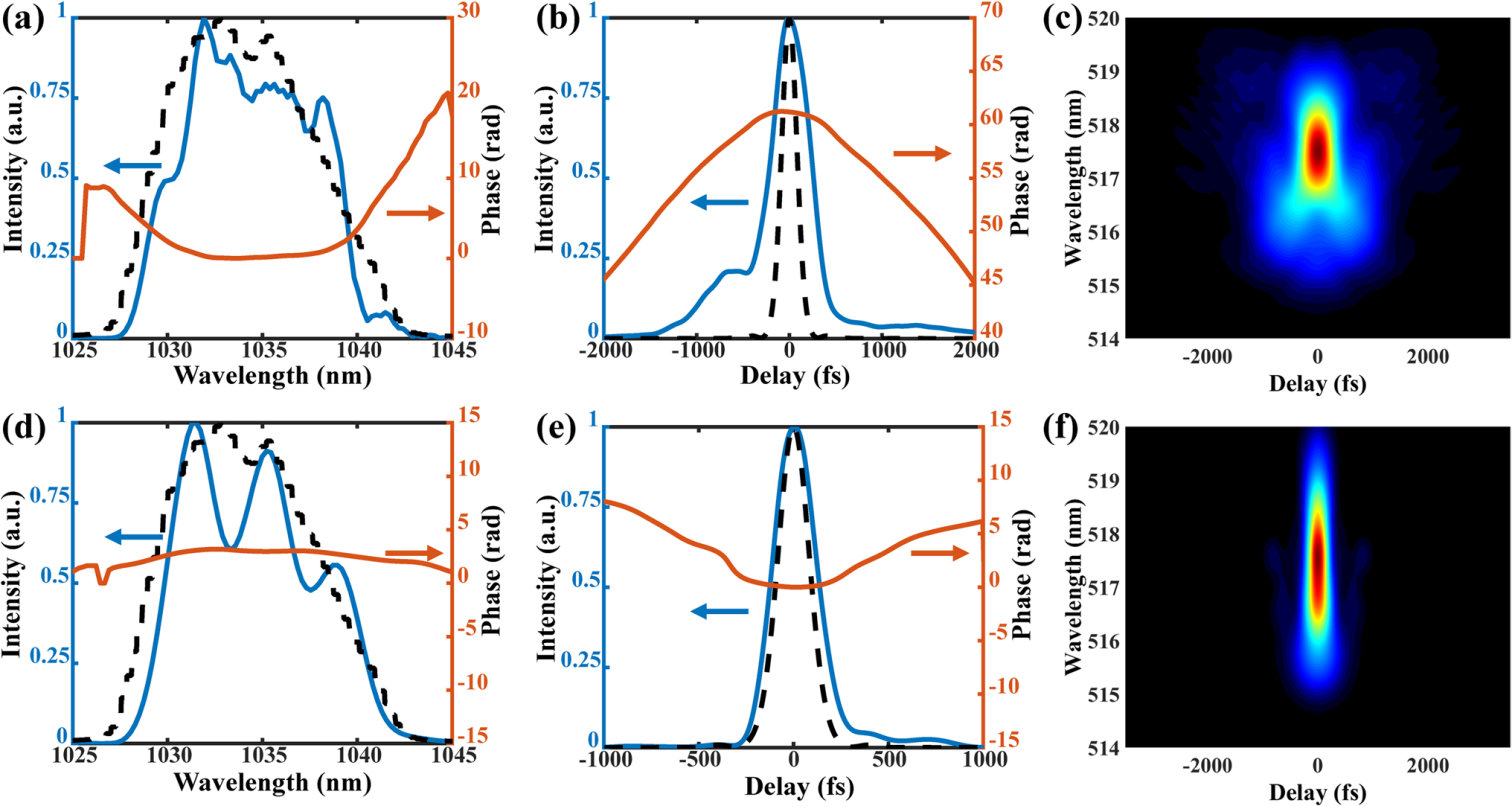
**a** Retrieved pulse envelope (blue) and phase (orange) of the compressed pulses before high-order dispersion compensation. The black dash is the Fourier transform-limit pulse calculated based on the measured spectrum. **b** Measured spectrum (black), retrieved spectrum (blue), and retrieved phase (orange) of the compressed pulses before high-order dispersion compensation. **c** Measured SHG-FROG trace before high-order dispersion compensation. **d** Retrieved pulse envelope (blue) and phase (orange) of the compressed pulses after high-order dispersion compensation. The black dash is the Fourier transform-limit pulse calculated based on the measured spectrum. **e** Measured spectrum (black), retrieved spectrum (blue), and retrieved phase (orange) of the compressed pulses after high-order dispersion compensation. **f** Measured SHG-FROG trace after high-order dispersion compensation

After phase-stabilizing the combined beam, the combined beam is transmitted to the compressor for pulse compression. To prevent potential damage to the gratings, the combined beam is recollimated to a diameter of 4 mm before entering the compressor. Through continuous optimization of the angles and spacing of the compression gratings elements, we achieve the shortest compression result of 488 fs, as illustrated in Fig. 3a. Nevertheless, an examination of the reconstructed spectral phase depicted in Fig. 3b reveals a substantial amount of uncompensated dispersion. Figure 3a further underscores this point, illustrating a notable disparity between the compressed pulse and the Fourier transform-limit pulse corresponding to the measured spectrum. Upon closer spectral phase analysis, it is ascertained that a residual group delay dispersion (GDD) of 570 fs^2^ remained uncompensated. Additionally, the values of third-order dispersion (TOD) at 1.34 × 10^–2^ ps^3^ and fourth-order dispersion (FOD) at 1.4 × 10^–3^ ps^4^ are left unaddressed. While the GDD is largely mitigated, the primary lingering issues reside in the third- and fourth-order dispersion.

While spectral shaping or post-compression methods can be employed to achieve further pulse duration compression, these techniques introduce increased system complexity and operational difficulties. Building upon the diagnostic results mentioned earlier, we employ TPSR to accurately compensate for high-order dispersion, resulting in a final optimized pulse duration of 260 fs, as illustrated in Fig. 3d. Figure 3e displays the reconstructed spectrum and phase. The fitting analysis of the phase reveals that the third-order dispersion and fourth-order dispersion are reduced to 1.2 × 10^–4^ ps^3^ and 2 × 10^–4^ ps^4^, respectively. FOD decrease by an order of magnitude, and TOD decrease by two orders of magnitude compared to the scenario without high-order dispersion compensation, representing a significant improvement. Simultaneously, it is worth noting that the energy of the main pulse shown in Fig. 3a and d increases from 65 to 92%, significantly improving the pulse quality. Due to the limited compensation accuracy of TPSR, it is evident that the compensation for high-order dispersion is incomplete. In our future work, we will also investigate approaches to completely compensate for these residual higher-order dispersions, aiming to further optimize the pulse duration. The average power of the combined beam after compression measures 403 W, which corresponds to a pulse energy of 504 μJ.

**Fig. 4:**
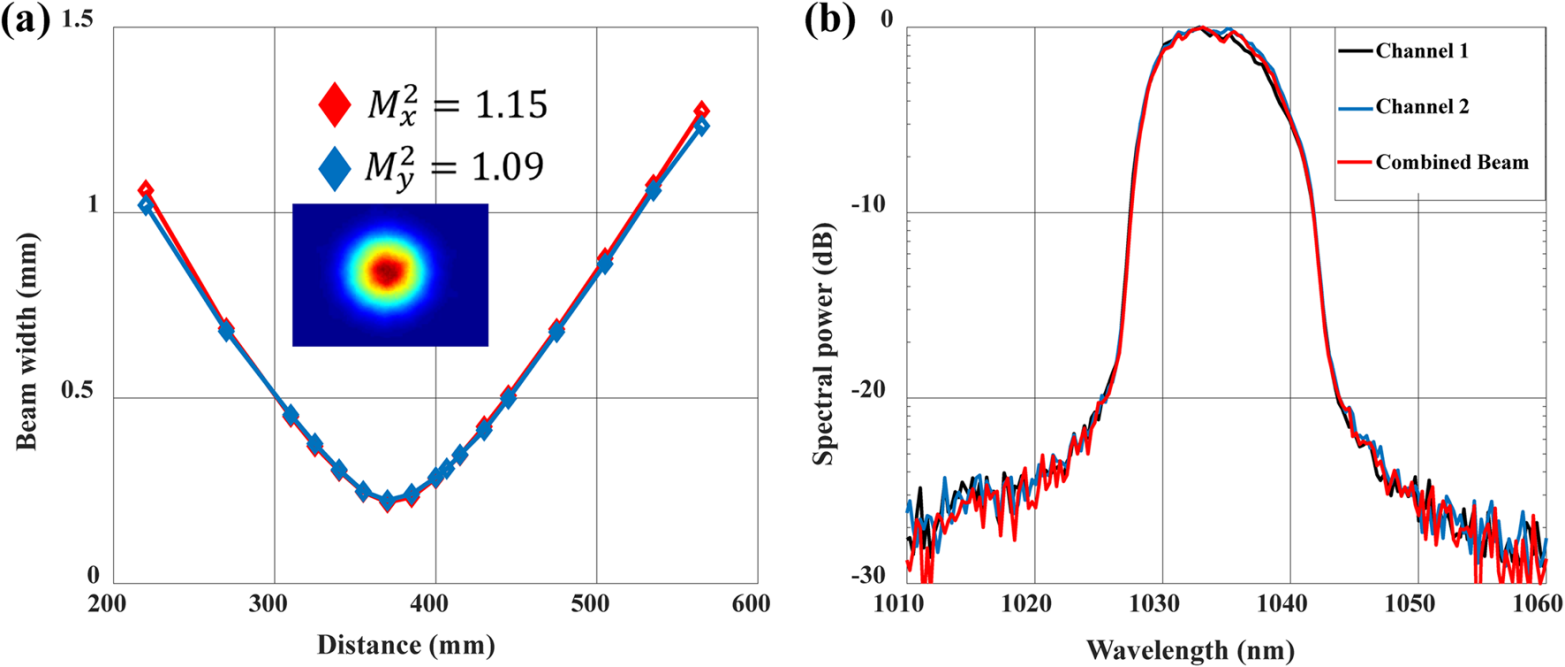
**a** *M*^2^ measurement of the combined beam. The intensity profile is shown in the inlay. **b** Spectrum of the individual channels and combined pulses

In addition, the beam quality *M*^2^ is measured by the 4*σ* method. As shown in Fig. 4a, the combined beam quality of the two axes almost reaches the diffraction limit with *M*^2^ < 1.2. Through meticulous power scaling control, it is apparent that the spectrum displays remarkable similarity between the two channels, as illustrated in Fig. 4b. Furthermore, the combined pulse spectrum closely emulates that of the individual channel, thereby establishing a robust groundwork for future improvements in combining efficiency.

## Conclusions

In summary, we present a high-power ultrafast fiber laser system that incorporates coherent beam combination of two amplifier channels. By employing coherent beam combination, we surpass the power limitation of single-channel rod fiber amplification and increase the system power to 433 W with a combined efficiency of 93%. The synergistic use of a tunable pulse stretcher (TPSR) enables precise compensation of residual higher-order dispersion. Together with the gratings compressor, we successfully optimize the pulse duration from 488 to 260 fs, enhancing the energy of the primary peak to 92%. The system delivers an average power of 403 W and a pulse energy of 504 μJ after the compressor. In addition, the CBC is executed with an average power exceeding 230 W per channel, simplifying system complexity and operational challenges. Leveraging these innovative rod-type photonic crystal fibers, it becomes feasible to generate femtosecond pulses with millijoule pulse energy and kilowatt average power using only four-channel coherent beam combination.

## Data Availability

The data that support the findings of this study are available from the corresponding author, upon reasonable request.
